# Short communication: TNF-α and IGF-1 regulates epigenetic mechanisms of HDAC2 and HDAC10

**DOI:** 10.1371/journal.pone.0263190

**Published:** 2022-02-10

**Authors:** Wanlin Jiang, Megan E. Block, Chandra S. Boosani

**Affiliations:** Department of Clinical and Translational Science, Creighton University School of Medicine, Omaha, Nebraska, United States of America; Duke University School of Medicine, UNITED STATES

## Abstract

Vascular restenosis often presents as a consequence of injury to the vessel wall, resulting from stenting and other interventional procedures. Such injury to the arteries induces proliferation of Vascular Smooth Muscle Cells (VSMCs), resulting in cellular hyperplasia and restenosis. We and others have previously reported de-novo production of different cytokines and growth factors such as Tumor Necrosis Factor Alpha (TNF-α) and Insulin like Growth Factor 1 (IGF-1), after vascular injury. As complex as it is, the profuse proliferation of VSMCs appears to be occurring due to several induced factors which initiate molecular mechanisms and exacerbate disease conditions. In many pathological events, the deleterious effects of TNF-α and IGF-1 in initiating disease mechanisms was reported. In the present work, we explored whether TNF-α and IGF-1 can regulate epigenetic mechanisms that promote proliferation of VSMCs. We investigated the mechanistic roles of proteins which can structurally interact with DNMT1 and initiate cellular pathways that promote proliferation of VSMCs. Our findings here, identify a novel molecular mechanism that is initiated by TNF-α and IGF-1. It was previously reported that DNMT1 expression is directly induced by TNF-α and IGF-1 treatment and increased/induced expression of DNMT1 causes silencing of genes that are essential to maintaining cellular homeostasis such as the tumor suppressor genes. We have earlier reported that TNF-α and IGF-1 treatment elevates DNMT1 expression in VSMCs and causes increased VSMC proliferation. However, the molecular mechanisms involved were not fully deciphered. Interestingly, in the present study we found that TNF-α and IGF-1 treatment failed to elevate DNMT1 expression levels in absence of HDAC2 and HDAC10. Also, while HDAC2 expression was not affected by HDAC10 knockdown, HDAC2 is essentially required for HDAC10 expression. Further, in TNF-α and IGF-1 induced epigenetic signaling mechanism, the expression of two important proteins EZH2 and PCNA seem to be regulated in an HDAC2-HDAC10 dependent manner. Our results show an inter-dependence of epigenetic mediators in inducing proliferation in VSMCs. To our knowledge, this is the first report that shows HDAC2 dependent expression of HDAC10, and suggests a novel mechanistic link between DNMT1, HDAC10 and HDAC2 that regulates EZH2 and PCNA to enhance cell proliferation of VSMCs which is the underlying cause for neointimal hyperplasia and restenosis.

## Introduction

Vascular occlusions in the coronary arteries are a serious concern for normal heart functions. Their occurrence in the coronary arteries would be alarming as they can cause ischemic heart disease and myocardial infarction [1,2]. The interventional procedure, Percutaneous transluminal coronary angioplasty (PTCA) followed by stenting is a common clinical practice performed to depress the atherosclerotic plaque and to restore luminal patency. However, PTCA itself can cause vascular smooth muscle cell (VSMC) proliferation, which leads to neointimal hyperplasia and stenosis [1]. Intra-vascular injury in the arteries causes endothelial denudation and damage to the intima during the interventional procedure. Subsequently, leukocyte infiltration and platelet accumulation occur which stimulates the production of inflammatory cytokines and growth factors, and this results in VSMC proliferation, migration, and phenotype switch causing neointimal hyperplasia, and within a few months, the medial layer thickens compromising the lumen of the artery [3]. To retain the vascular lumen patent, stents are often deployed at the site of PTCA. However, stenting often could result in intimal hyperplasia and in-stent restenosis, and to avoid in-stent restenosis, several modifications and improvements in stents were made. Disappointing is that even the recent and most advanced biodegradable/bio-absorbable stents also failed in clinical trials due to secondary effects [4,5]. In this imminent need to prevent the incidence of restenosis after PTCA or stenting, efforts were heightened to identify novel molecular targets, disease mechanisms and new approaches for such post procedural complications.

Several de-novo induced epigenetic mechanisms have been reported which play a significant role in pathological progression of different human diseases, including cardiovascular diseases [6–21]. DNA Methyltransferases (DNMTs) and Histone deacetylases (HDACs) are two well characterized group of enzymes which predominately regulate epigenetic mechanisms occurring in living cells. During normal cell growth and division, HDACs and DNMTs ensure tight control on temporal and spatial expression of inherited genes.

Control over gene regulation by DNMTs occurs primarily through methylation of the genomic DNA. Different isoforms of DNMTs were identified which have unique preferences on DNA sequences they methylate. Although DNMTs are involved in maintaining cellular homeostasis, it is their aberrant activities that alters the normal cellular functions, leading to disease developmental and abnormalities. The pathological role of DNMT1 in promoting coronary restenosis was recently reported by us. We showed that induced expression of DNMT1 inhibits the tumor suppressor protein “Suppressor of Cytokine Signaling”, SOCS3, in VSMCs. The expression of DNMT1 was found to be regulated by two different pathways that independently drive initiation and progression of coronary restenosis [1,22–24].

HDACs are another group of epigenetic regulators whose functions were discernably proven to be important in invoking disease-causing molecular mechanisms. HDACs are a unique class of enzymes which remove acetyl group on amino acids, mainly the lysine residues, of histone proteins which wrap the DNA. This deacetylation process induces a more tightly coiled state of the chromatin and causes gene silencing [25]. One of the isoforms of HDACs, HDAC2 was shown to induce the expression of oncogenes such as Myc to promote cell proliferation. HDAC2 was also reported to suppress the expression of tumor suppressor genes such as p53 and p21. in addition, there is ample evidence in the literature which supports a prominent role of HDAC2 in pathological conditions where cell proliferation is often enhanced. In VSMCs, HDAC2 was reported to prevent the expression of the tumor suppressor protein p21, indicating its aggressive role in enhancing cardiovascular complications [26]. Recent reports have shown that gene expression regulated by the complex interactions between DNMT1, HDAC2 and PCNA, are required to prevent proliferation of HEK293 cells [27,28]. Interpretations from the molecular structure of DNMT1 further revealed the presence of several unique regions in DNMT1 with which it potentially interacts with other proteins, such as PCNA, HDAC1, HDAC2, and EZH2 [28].

Our previous studies have identified two different molecular pathways of DNMT1 that work independently to repress the expression of a tumor suppressor protein, SOCS3, and promotes the development of neointimal hyperplasia [1,22–24]. Our continued research in this direction led us to explore interacting proteins and pathways which regulate DNMT1 activity during restenosis. Further, we also reported that when DNMT1 expression was inhibited in VSMCs, its absence was supplemented by another isoform of DNMT, DNMT3a [24]. Since DNA methylation is a key mechanism by which gene expression is regulated in a controlled manner, the induction of subsidiary methylases, in absence of DNMT1, seems to be required for cell survival. On these lines, autophagy is undoubtedly a very common cellular function which helps cell survival through recycling intra-cellular constituents. It was reported that a specific isoform of Histone Deacetylase protein, HDAC10, protects the cells from undergoing apoptosis by initiating cellular mechanisms of autophagy [38–44]. Interestingly, the class I histone deacetylase HDAC2, was identified to interact with HDAC10, which is a class II isoform [29]. Although the expression of HDAC2 seem to correlate with HDAC10 expression in certain diseases such as liver cancer and gastric cancer, no definitive interactions were reported between the two, or their inter-dependent effects on gene regulations [30,31].

In the present study, we explored the functional interactions between the epigenetic mediators that play a key role in promoting restenosis. The results presented here support previous publications and suggest the mediation of HDAC10 in indirectly altering the cellular functions which are regulated by the molecular signaling initiated by DNMT1 and HDAC2. The novel findings presented here highlight the significance of HDACs and DNMT1 in regulating proliferation of VSMCs, which leads to the development of neointimal hyperplasia and restenosis, in-vivo.

## Materials and methods

DMEM, SMCM, Opti-MEM medium, TNF-α, IGF-1, RIPA buffer, protease-phosphatase inhibitors, BCA reagent, Laemelli buffer, beta-mercaptoethanol, 10% SDS PAGE gels, TBST buffer, Trypsin-EDTA, PBS, Collagenase, FBS, Antibiotic solution, Romidepsin, siRNAs, Lipofectamine RNAiMax, BrdU kit, PVDF membrane, BSA, ECL reagent, TGE buffer, Antibodies (DNMT1, EZH2, HDAC2, HDAC10, PCNA, GAPDH, HRP secondary).

### VSMC isolation and culture

Swine hearts from locally raised farm pigs (food animals) were purchased from the local butcher plant (slaughterhouse). Whole hearts were collected in DMEM medium, and about one-inch length left circumflex artery from the swine heart was excised and thoroughly rinsed in fresh DMEM medium. The artery was cut open with sterile scissors and after removing the adventitia and scraping off the endothelial layer, the smooth muscle tissue was finely chopped with a sterile scalpel-blade. The minced tissue was transferred to 0.025% Trypsin-EDTA solution and incubated at 37°C for 45 minutes in a total volume of about 10 ml. Subsequently, the trypsin digested sample was centrifuged at 1500 rpm for 5 minutes and the pellet was suspended in about 10 ml of 0.25% collagenase solution (prepared in DMEM medium). After incubating for 4 hrs at 37°C, the collagenase digest was centrifuged for 5 minutes at 1500 rpm and the pellet was suspended in 10 ml of smooth muscle cell complete medium (SMCM). This cell suspension was then filtered through 40 micron cell strainer and about 5 ml of the flow through was transferred into a T25 flask. The flask was incubated at 37°C overnight in a humidified incubator that was maintained with 5% CO_2_. The next day, medium was replaced with fresh SMCM medium and the cells were continued to grow in the incubator. Between 7 to 10 days of culture, VSMCs growth appeared in clusters. The cells were trypsinized and about one million cells were transferred to a T25 flask. This initial culture was considered as passage 1, and when reached about 70% confluence, they were trypsinized and sub-cultured again by splitting them in 1:3 ratio. VSMCs that were cultured up to passage 3 or 4 were used for the studies outlined here.

### VSMC transfection and treatment

When VSMCs reached to about 70% confluence, they were transfected with corresponding siRNA using Lipofectamine RNAiMax. Briefly, in an eppendorf tube, about 30 picomoles of siRNA was diluted in 150 μl of Opti-MEM medium. In another eppendorf tube 9 μl of Lipofectamine RNAiMax was diluted in 150 μl of Opti-MEM medium. Both the tubes were incubated for about five minutes at room temperature in the biosafety cabinet and then mixed to prepare transfection mix. Subsequently, the transfection mix was incubated for additional 30 minutes at room temperature in the biosafety cabinet. To about 250K VSMCs cultured in SMCM medium in each well of a 6-well plate, the above transfection mix was added and incubated overnight in the incubator maintained at 37°C with 5% CO_2_. After about 16 hrs, the transfection mix was replaced with fresh SMCM medium and the cells were cultured for additional 24 hrs. Both transfected and un-transfected VSMCs were treated with 100 ng/ml concentrations of TNF-α or IGF-1 or both together for 24 hrs. The medium supernatant was then aspirated, and the cells were briefly washed with warm PBS before lysing them in RIPA buffer supplemented with protease and phosphatase inhibitors. After adding the lysis buffer, the plates were incubated on ice for about 5 minutes. The lysate was then pooled and collected with a cell scraper. Finally, the lysate was centrifuged at 12000 rpm for 10 minutes in a microfuge maintained at 4°C. The protein content in the lysates were then mixed with BCA reagent and incubated at 37°C for one hour. The amount of protein in each lysate was quantified using a spectrophotometer by measuring the absorbance at 560 nm.

### Western blotting and protein detection using chemiluminescent assay

About 30 μg of protein lysate from each sample was used for western blotting. The lysate was mixed with 4x Laemelli buffer containing beta-mercaptoethanol and incubated at 95°C for 5 minutes for denaturation. Samples were then resolved on 10% SDS-PAGE gels at 140 volts for about 90 minutes or until the dye front leaves the gel from the bottom side. Proteins resolved in the gel were transferred onto the PVDF membrane in a transfer unit that was maintained at a constant voltage of 50 volts. The western transfer was continued overnight at 4°C. The next day, the blotted PVDF membrane was blocked for one hour in TBST buffer containing 5% BSA, and the membrane was probed with primary antibody diluted at 1 μg/ml in TBST buffer. Probing with the primary antibody was performed overnight at 4°C on a rocker. Subsequently, the membrane was washed three times for five minutes each in TBST buffer at room temperature on a rocker. The PVDF membrane was then probed with HRP conjugated secondary antibody that was diluted at 0.5 μg/ml in TBST, for one hour at room temperature on a rocker. The membrane was then washed for three times in TBST for five minutes each and then incubated with 2 ml of ECL reagent mix for one minute. Proteins corresponding to the primary antibodies were detected using “Bio-Rad Chemidoc” image capture and analyzer.

### Cell proliferation assay

The BrdU incorporation assay was performed to assess VSMC proliferation using BrdU ELISA kit (Roche Applied Science). VSMCs (about 2500/well) were cultured in 96-well plates and treated with TNF-α or IGF-1 or both together at 100 ng/ml each. After 24 of treatment the cells were treated with 10 mM BrdU solution and incubated overnight at 37°C. The supernatant was discarded, and the cells were air dried for fixation. The cellular DNA was denatured by adding FixDenat solution and incubating it for 30 min at room temperature. Mouse anti-BrdU monoclonal antibody conjugated with peroxidase was added to each well of the 96-well plate and incubated at room temperature for 2 hrs. Subsequently, Tetramethylbenzidine was added and the cells were incubated briefly for 5 min at room temperature. Finally, the absorbance in the sample supernatant was measured using a microplate reader (PerkinElmer) that was set at 370 nm absorbance.

### Statistical analysis

The band intensities in different western blots obtained from different exposures were quantified using LI-COR image studio software. Data was analyzed using GraphPad Prism version 7.0. Unpaired student’s t-test was used to determine differences between groups. Multiple group comparisons were made using one-way ANOVA with Turkey’s post-hoc tests. Values are expressed as mean ± SEM (standard error of the mean). A p-value of < 0.05 was considered significant in comparing statistical difference between the groups.

## Results

### Effects of HDAC2 silencing on DNMT1 and EZH2 expression

**Fig 1:**
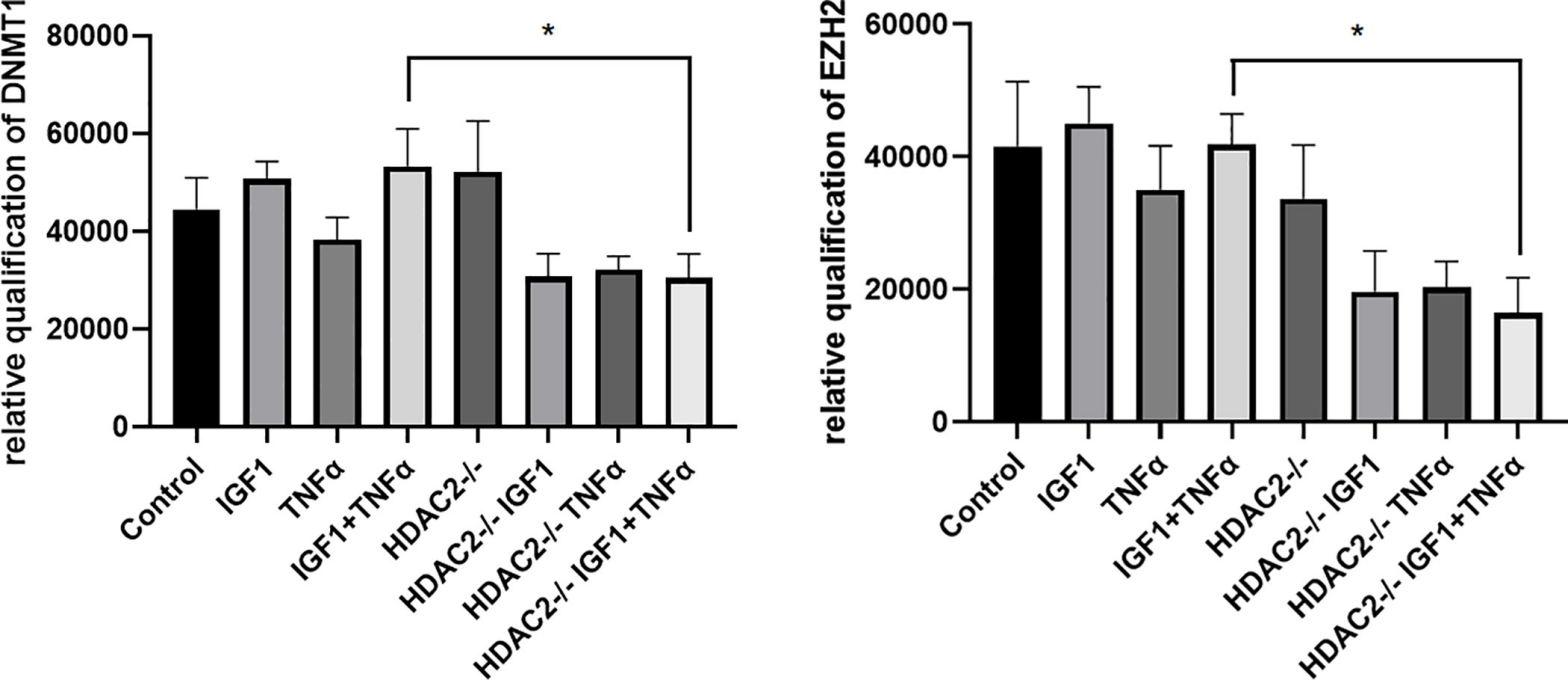
Effects of HDAC2 knockdown on the expression of epigenetic markers. Figure is a western blot analysis of the expression of important epigenetic mediators that are presumably located upstream and downstream of HDAC2. Primary VSMCs were transfected with or without siRNA specific for HDAC2 and treated with or without 100 ng/ml IGF-1 or 100 ng/ml TNF-α or both together. Top panel shows the expression of DNMT1 and the middle panel shows the expression of EZH2. Expression of GAPDH was shown as loading control.

VSMCs that were cultured to about 70% confluence were transfected with siRNA that is specific to HDAC2. After 48 hrs of transfection, the cells were treated for 24 hours, with or without TNF-α and IGF-1 together at 100 ng/ml concentration. After 72 hours of transfection, total protein was isolated from the transfected and untransfected cells and about 30 ug of total protein was subjected to electrophoresis in 10% SDS-PAGE gels and subsequently, western blotting was performed. The blots were probed overnight with primary antibodies that detect DNMT1 or EZH2. In Fig 1, the top panel shows the expression of DNMT1 in control and HDAC2 knockdown VSMCs that were treated with or without TNF-α and IGF-1. The expression of DNMT1 was seen reduced in HDAC2 knockdown cells when compared to the untransfected cells. Importantly, the TNF-α and IGF-1 induced expression of DNMT1 which was prominently seen in untransfected VSMCs (lane 4) was drastically reduced in HDAC2 knockdown cells that were also treated with TNF-α and IGF-1 (lane 8). The middle panel in Fig 1 shows the expression of EZH2 in control and HDAC2 knockdown VSMCs. Similar to the expression pattern observed with DNMT1 expression in the top panel of this figure, the expression of EZH2 was also seen drastically reduced in the HDAC2 knockdown cells that were treated with TNF-α and IGF-1. Expression of the housekeeping protein GAPDH was shown as loading control.

### Treatment with Romidepsin affects the expression of epigenetic mediators

**Fig 2:**
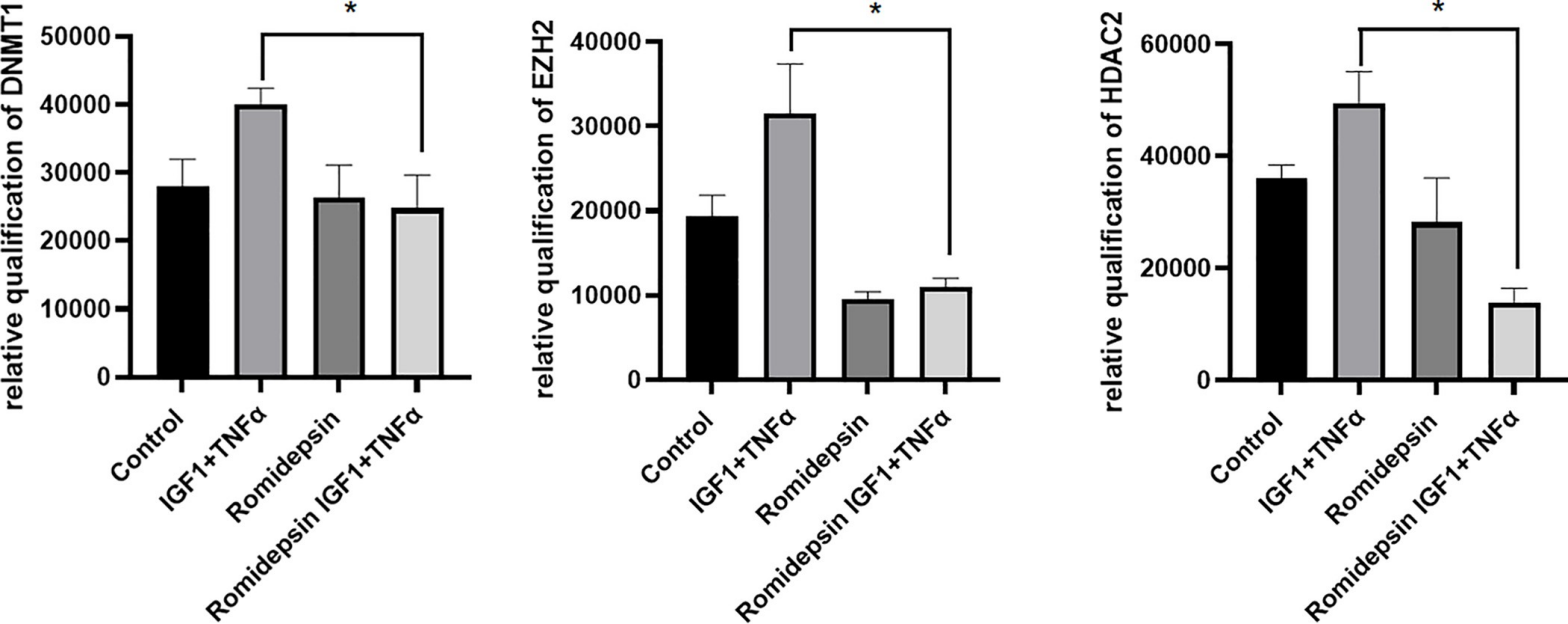
Inhibitory effects of Romidepsin on VSMCs treated with or without IGF-1 and TNF-α. Figure is a western blot showing changes in the expression of DNMT1, EZH2 and HDAC2 in primary VSMCs that were treated with 50 nM Romidepsin and 100 ng/ml of both IGF-1 and TNF-α. Expression of GAPDH was tested as a loading control. VSMCs that were not treated with Romidepsin or IGF-1 and TNF-α were shown as control.

Romidepsin is a specific inhibitor of HDAC2. We evaluated whether inhibition of HDAC2 by Romidepsin treatment would have any effect on DNMT1 and EZH2 expression. VSMCs were cultured to 70% confluence and serum starved overnight. Subsequently, they were treated with Romidepsin at 50 nM concentration for 20 hrs. TNF-α and IGF-1 treatment was initiated 2 hrs after addition of Romidepsin to the cultured cells. After 24 hrs, about 30 ug of total protein from the cell lysate was subjected to electrophoresis and western blotting. The expression of DNMT1 and EZH2 were evaluated using corresponding antibodies. The top panel in Fig 2, shows the expression of DNMT1 in VSMCs that were treated with or without Romidepsin and with or without TNF-α and IGF-1 together. The expression of DNMT1 induced by TNF-α and IGF-1, was seen drastically reduced with Romidepsin treatment. The middle panel in Fig 2, shows the expression of EZH2 whose expression was also seen reduced due to treatment with Romidepsin, and is independent of TNF-α and IGF-1 treatment. Fig 2 also shows the effect of Romidepsin on HDAC2 expression in VSMCs in different treatment conditions stated above. The bottom panel shows expression of the housekeeping protein GAPDH, shown as loading control.

### PCNA expression in HDAC2 and HDAC10 knockdown VSMCs

**Fig 3:**
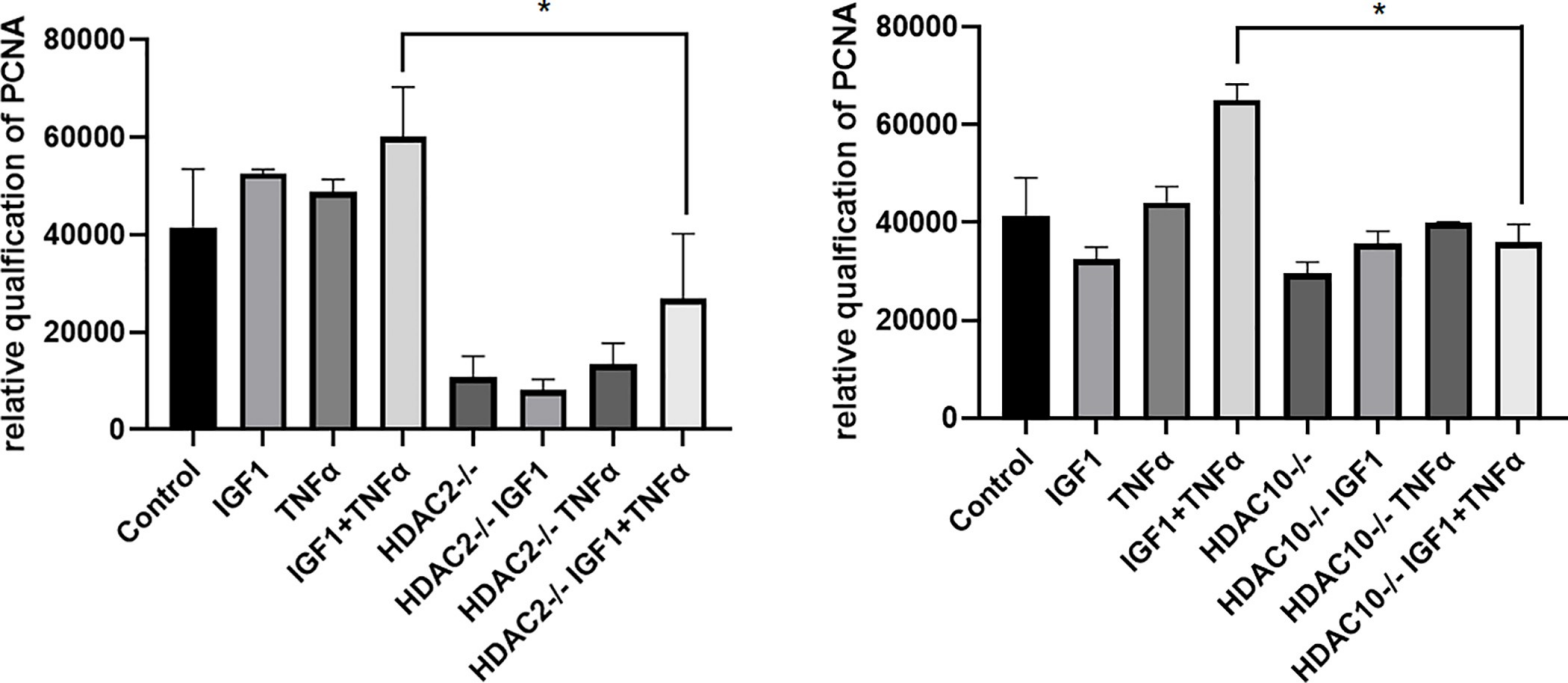
Effects of HDAC2 and HDAC10 silencing on the expression of cell proliferation marker, PCNA. Figure is a western blot showing the expression of the cell proliferation marker, PCNA. Primary VSMCs were transfected with or without siRNA specific for HDAC2 or HDAC10 and treated with or without 100 ng/ml IGF-1 or 100 ng/ml TNF-α or both together. Top panel shows the expression of PCNA in control and HDAC2 knockdown cells and the bottom panel shows the expression of PCNA in HDAC10 knockdown cells. Expression of GAPDH was shown as loading control in both top and bottom panels.

A more representative marker for active cell proliferation is PCNA, whose altered expression was evaluated in HDAC2 or HDAC10 knockdown cells that were treated with or without TNF-α and IGF-1. VSMCs were cultured to 70% confluence and transfected with or without siRNA specific for HDAC2 or HDAC10. After 48 hrs of transfection, they were treated with or without TNF-α and IGF-1 for 24 hrs. After isolating the cell lysate, about 30 ug of total protein from each lysate was subjected to SDS-PAGE electrophoresis on 10% Acrylamide gels, and western blotting was subsequently performed. Blots were probed with primary antibodies that detect PCNA. The top panel in Fig 3, shows the expression of PCNA in control and HDAC2 knockdown cells that were treated with or without TNF-α and/or IGF-1. Independent of TNF-α and/or IGF-1 treatment, drastic reduction in the expression of PCNA was observed in HDAC2 knockdown cells. The bottom panel in Fig 3, shows the expression of PCNA in control and HDAC10 knockdown cells that were treated with or without TNF-α and/or IGF-1. The expression of PCNA appears to be marginally affected when compared between the control and HDAC10 knockdown cells. However, compared to untransfected control cells, PCNA expression was seen substantially reduced in HDAC10 knockdown VSMCs in presence of TNF-α and IGF-1 (protein band in lane 4 compared with lane 8). Western blots were probed with GAPDH antibodies in the same lysates were shown as loading control.

### Inter-dependent expression of HDAC2 and HDAC10

**Fig 4:**
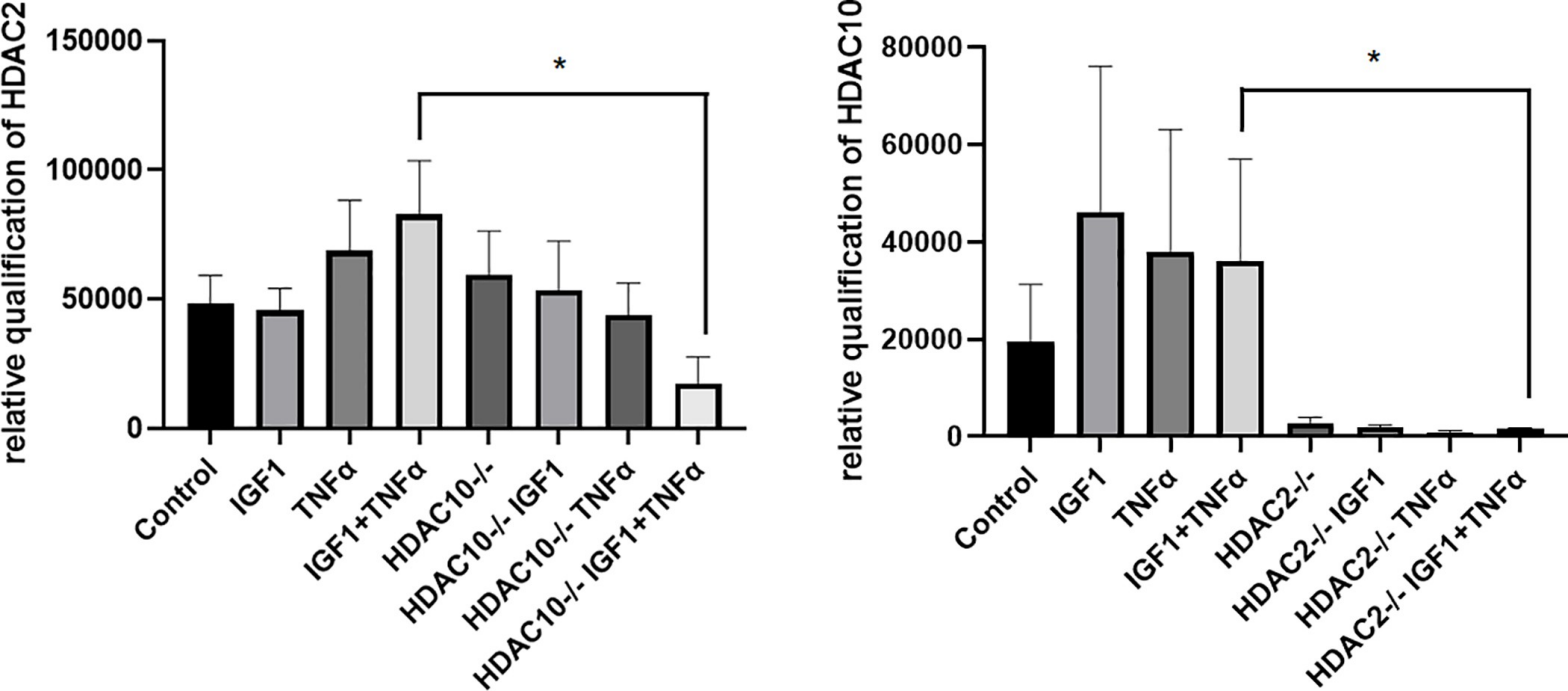
Analysis of HDAC2 and HDAC10 interdependent expression in primary VSMCs. Figure is a western blot showing the expression of HDAC2 and HDAC10 along with GAPDH as loading control. Top panel shows the expression of HDAC2 in control and HDAC10 knockdown cells that were treated with or without 100 ng/ml IGF-1 or 100 ng/ml TNF-α or both together. Bottom panel shows the expression of HDAC10 in control and HDAC2 knockdown cells that were treated with or without 100 ng/ml IGF-1 or 100 ng/ml TNF-α or both together.

To know whether HDAC10 expression is regulated downstream to HDAC2, VSMCs that were cultured to 70% confluence were transfected with or without siRNA corresponding to HDAC2 or HDAC10, independently. After 48 hrs of transfection, the cells were treated with or without TNF-α and/or IGF-1, and after 72 hrs of transfection, cell were lysed and about 30 ug of total protein from the cell lysates was subjected to electrophoresis in 10% SDS-PAGE gels. Subsequently, western blotting was performed to detect the presence of HDAC2 or HDAC10 proteins. The top panel in Fig 4, shows the expression of HDAC2 in HDAC10 knockdown cells. Expression of HDAC2 was seen decreased in the TNF-α and IGF-1 treated group when compared between control and HDAC2 knockdown cells (protein bands in lane 4 compared with lane 8). The bottom panel in Fig 4, shows the expression of HDAC10 in HDAC2 knockdown cells. Independent of the TNF-α and IGF-1 treatment, the expression of HDAC10 was seen inhibited in all HDAC2 knockdown cells.

### Effects of HDAC10 silencing on EZH2 and DNMT1 expression

**Fig 5:**
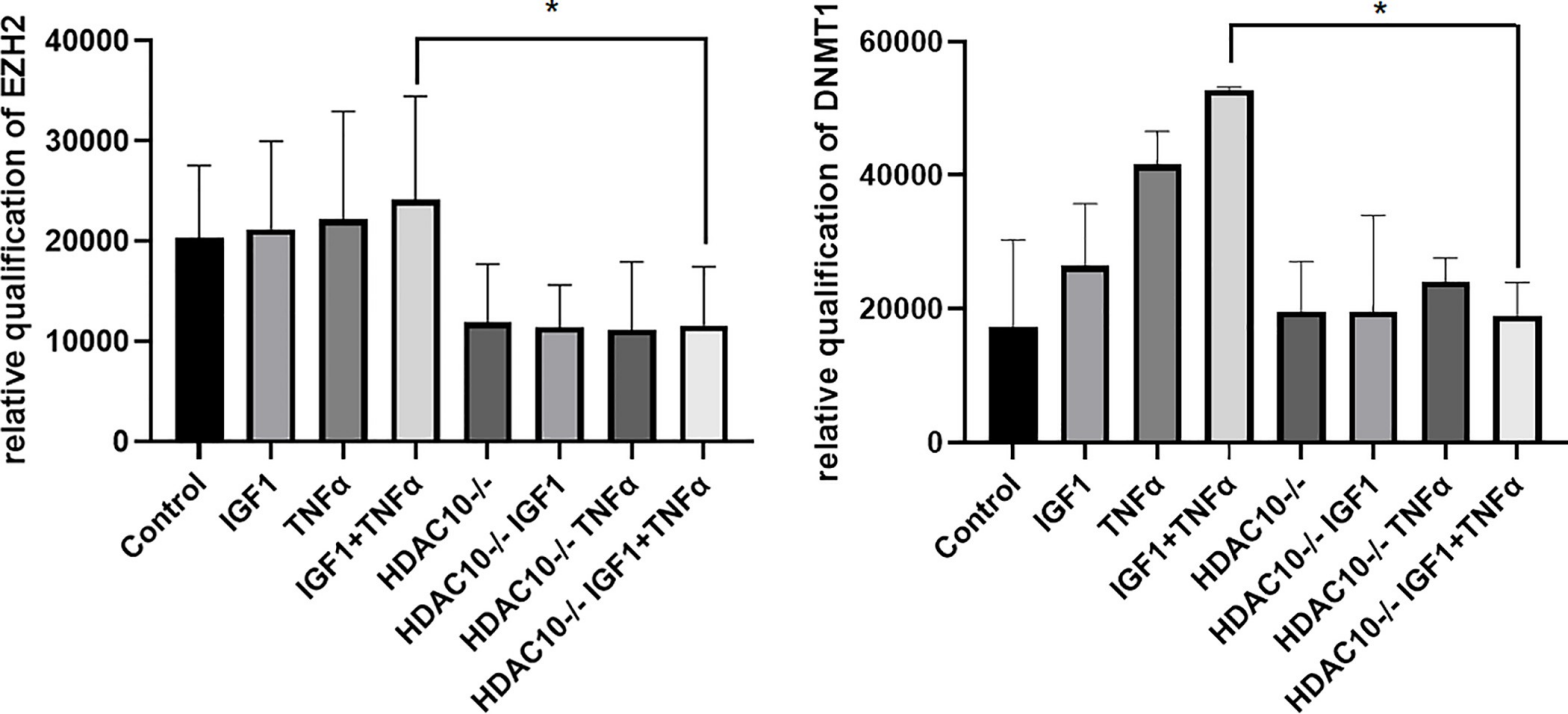
Effects of HDAC10 knockdown on the expression of epigenetic markers. Figure is a western blot showing the expression of important epigenetic mediators that presumably function upstream and downstream to HDAC2. Primary VSMCs were transfected with or without siRNA specific for HDAC10 and treated with or without 100 ng/ml IGF-1 or 100 ng/ml TNF-α or both together. Top panel shows the expression of EZH2 and the middle panel shows the expression of DNMT1. Expression of GAPDH was shown as loading control in the bottom panel.

VSMCs that were cultured to 70% confluence were transfected with or without siRNA that specifically targets HDAC10. After 48 hrs of transfection, the cells were treated with or without TNF-α and IGF-1 together. After 72 hours of transfection, the total protein from cell lysate was collected and about 30 ug of protein from each lysate was used for SDS-PAGE electrophoresis. Subsequently, western blotting was performed, and the blots were probed overnight with antibodies specific to EZH2 and DNMT1. The top panel in Fig 5, shows the expression of EZH2. Decreased expression of EZH2 was seen in HDAC10 knockdown cells which appears to be affected independent of TNF-α and/or IGF-1 treatment. The middle panel in Fig 5, shows the expression of DNMT1 in the same blot, which appears to be affected by HDAC10 knockdown. Importantly, the TNF-α and IGF-1 treatment induced expression of DNMT1 (lane 4), was seen drastically reduced when HDAC10 expression was silenced (lane 8). The bottom panel shows the expression of GAPDH as loading control.

### Cell proliferation in HDAC2 and HDAC10 knockdown VSMCs

**Fig 6:**
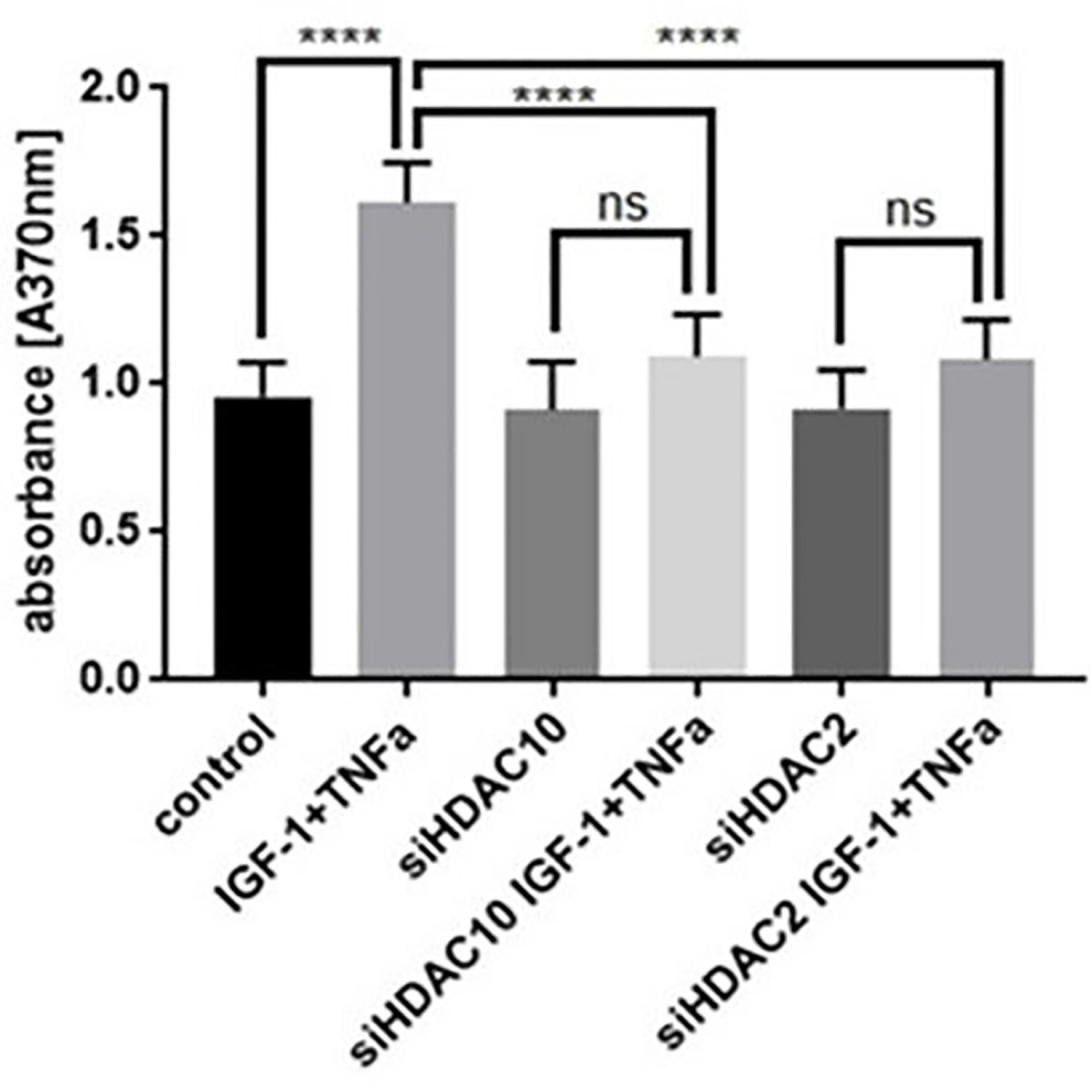
Cell proliferation in VSMCs transfected with siRNA-HDAC10 or siRNA-HDAC2 and treated with or without IGF-1 and TNF-α. The primary VSMCs were transfected with siRNA specific for HDAC10 or HDAC2 and treated with 100 ng/ml IGF-1 and TNF-α. Cell proliferation was assessed using BrdU staining. Figure shows data from six independent experiments. Data was presented as mean with SEM in the error bars. A p-value of <0.05 was considered as statistically significant. The analyzed p value was p < 0.0001 ****).

After 24 hrs after transfection, control and siRNA knockdown VSMCs were seeded in a 96-well plate and treated with or without TNF-α and IGF-1 for 24 hrs. Cell proliferation in VSMCs was assessed using BrdU staining kit following the manufacturers protocol. The extent of VSMC proliferation was assessed by measuring the absorbance at 370 nm, where increase in absorbance was directly correlated with increased rate of cell proliferation. As seen in Fig 6, control VSMCs that were treated with TNF-α and IGF-1 showed the highest absorbance. In HDAC2 and HDAC10 knockdown cells, the absorbance was similar to control cells. The HDAC2 and HDAC10 knockdown cells that were treated with TNF-α and IGF-1 showed no significant increase in cell proliferation when compared to the corresponding control groups.

## Discussion

Previously, we reported two distinct molecular mechanisms associated with proliferation of VSMCs, Jak-Stat signaling and DNMT1 mediated epigenetic mechanisms [1,22,32]. These two mechanisms were reported by us and also by others in the field, as to be involved in promoting vascular restenosis occurring as a consequence of interventional procedures such as cardiac catheterization. In both these molecular mechanisms, induced expression of DNMT1 observed in presence of both TNF-α and IGF-1 treatment, seem to function as an active epigenetic modulator promoting profuse proliferation of VSMCs. It is to be noted that TNF-α and IGF-1 each have their own independent molecular mechanisms of actions however, it is the combined effect of TNF-α and IGF-1 which when present together, induces the expression of DNMT1. Presumably, the two molecular pathways cross talk to initiate a disease specific pathway. Our group and others have been investigating this unique combined effect of TNF-α and IGF-1 in different diseases. In several pathological conditions, DNMT1 induced cell proliferation was reported, and use of different DNMT1 inhibitors were evaluated in the pre-clinical settings. With the interest to prevent active cell proliferation, FDA approved 5-Aza-Cytidine which is a non-specific inhibitor of DNMT1 and is currently used in the treatment of select human diseases [33–37].

Recently, it was shown that a specific isoform of Histone Deacetylase protein, HDAC10, protects the cells from undergoing apoptosis by initiating cellular mechanisms of autophagy [38–44]. However, there is no sufficient evidence in the literature to delineate how the two prominent epigenetic mechanisms, DNA methylation and DNA acetylation, crosstalk to induce or regulate VSMC proliferation in pathological conditions. Thus, it became our interest to explore the possible molecular mechanisms that regulate two major epigenetic pathways that can promote profuse proliferation of VSMCs.

In our recent reports we showed that the combined treatment with TNF-α and IGF-1 induces the expression of DNMT1 and facilitates profuse proliferation of VSMCs [1,22–24]. One of the main molecular functions of DNMT1 is to inhibit tumor suppressor proteins such as p53 and SOCS3, to facilitate active cell proliferation [45–47]. It was previously reported that protein-protein interactions between HDAC2 and DNMT1 are essential to prevent transcription of the methylated DNA sequences [48,49]. The presence of HDAC2 interacting region on DNMT1 further suggests that gene silencing through methylation in the CpG islands by DNMT1 may get effectuated by the protein-protein interactions between DNMT1 and HDAC2.

In the present study, to understand the significance of HDAC2 in DNMT1 mediated gene silencing, we transfected VSMCs with siRNA specific for HDAC2 and evaluated the expression of DNMT1 in them. As seen in the top panel of a 1, knockdown of HDAC2 did not had any effect on DNMT1 expression (lane 1 compared with lane 5). However, when HDAC2 knockdown cells were treated with TNF-α or IGF-1 or both together, the expression of DNMT1 was seen decreased (lanes 2, 3 and 4 were compared with lanes 6, 7 and 8, respectively). It was interesting to note that TNF-α and IGF-1 failed to induce DNMT1 expression in absence of HDAC2 (lane 4 compared with lane 8). These observations suggest that HDAC2 may be required for TNF-α and IGF-1 induced epigenetic mechanisms. Similar effects of HDAC2 knockdown were also seen on EZH2 expression. HDAC2 knockdown alone seem to have no effect on EZH2 expression (Fig 1, middle panel, lane 1 compared with lane 5), but when HDAC2 knockdown cells were treated with TNF-α or IGF-1 or both together, the expression of EZH2 was seen decreased (lanes 2, 3 and 4 were compared with lanes 6, 7 and 8, respectively). In an interesting recent study, inhibition or knockdown of EZH2 expression was shown to induce cell death in VSMCs [50]. Further, in the same study overexpression of EZH2 was shown to enhance cell growth. Notably, neither cell proliferation nor induced apoptotic mechanisms were observed but instead, increased number of autophagic vacuoles were reported [55]. In the present study, we found that expression of EZH2 was not affected in HDAC2 knockdown cells that were not treated with TNFα or IGF1. Our results corroborate with the above published reports and suggests that EZH2 mediated cell protection may occur from the mechanisms that induce autophagy. Thus it could be hypothesized that there exists an indirect mechanism by which TNF-α and IGF-1 induces the expression of DNMT1, and it is very likely be mediated by HDAC2.

In attempts to elucidate the inter-dependencies between HDAC2 and DNMT1, we treated VSMCs with HDAC2 specific inhibitor, Romidepsin. It was earlier reported that by producing zinc binding thiols, Romidepsin prevents zinc dependent activity of histone deacetylases [51–54]. In our study, when VSMCs were treated with Romidepsin we observed inhibition of HDAC2, EZH2 and DNMT1 (Fig 2). Since Romidepsin specifically inhibits HDAC2, and as we observed decreased expression of EZH2 and DNMT1 with Romidepsin treatment, an essential role of HDAC2 in regulating the expression of EZH2 and DNMT1 in VSMCs can be inferred. Further, it is to be noted that the effects of Romidepsin on EZH2 and DNMT1 were independent of the treatment with TNF-α and IGF-1. These correlations and interpretations also imply indirect molecular functions of TNF-α and IGF-1 in regulating epigenetic mechanisms.

The N-terminal region of DNMT1 harbors a specific domain with which it interacts with PCNA, and since DNMT1 expression is regulated by HDAC2, we evaluated the expression of PCNA in VSMCs that were transfected with HDAC2 specific siRNA. As seen in the top panel of Fig 3, knockdown of HDAC2 in VSMCs inhibited the expression of PCNA. Recently, it was shown that TNF-α treatment elevates the levels of HDAC2 in cardiac muscle cells to cause mitochondrial dysfunction, without affecting HDAC10 expression [55]. Thus, HDAC10 expression is possibly not affected by treatment with TNF-α. Since EZH2 and HDAC10 are both involved in regulating cellular autophagy, we wanted to investigate if HDAC10 deletion would have any effect on PCNA or is influenced by TNF-α and/or IGF-1 treatment. Accordingly, we transfected VSMCs with HDAC10 specific siRNA, and evaluated the expression levels of PCNA in presence or absence of TNF-α and/or IGF-1 (Fig 3, lower panel). Increased PCNA expression due to TNF-α and IGF-1 treatment, was seen drastically reduced in VSMCs when HDAC10 expression was silenced (Fig 3 lower pane, expression in lane 4 compared with lane 8). These observations support molecular cross-talk between TNF-α and IGF-1 induced mechanisms that regulate indirect epigenetic pathways associated with VSMC proliferation.

The above results, and presence of different protein interacting domains on the N-terminal region of DNMT1, suggests a unique DNMT1 and HDAC2 mediated epigenetic mechanism. TNF-α was previously shown to induce the expression of HDAC2 and not HDAC10 [55]. Thus, the effects of the treatment with TNF-α and/or IGF-1, was tested in HDAC2 and HDAC10 knockdown VSMCs, independently. As seen in the top panel of Fig 4, TNF-α and IGF-1 induced expression of HDAC2 was inhibited when HDAC10 expression was silenced (lane 4 compared to lane 8). Similarly, we transfected VSMCs with HDAC2 specific siRNA, treated with or without TNF-α and/or IGF-1, and looked for the expression of HDAC10 in them. Interestingly, when HDAC2 expression was silenced, the expression of HDAC10 was completely inhibited in all HDAC2 knockdown cells, and this was seen independent of TNF-α and/or IGF-1 treatment (Fig 4, bottom panel). These findings suggest that the expression of HDAC2 is independent of HDAC10, but HDAC10 expression is dependent on HDAC2. These findings also indicate that HDAC10 may function downstream to HDAC2 in the epigenetic pathway.

Since HDAC10 and EZH2 are both the regulators of autophagy, it would be logical to evaluate whether HDAC10 has any direct effect on EZH2 and also DNMT1 expression. Accordingly, we tested the effects of HDAC10 knockdown on the expression of EZH2 and DNMT1 in VSMCs. As seen in the top panel of Fig 5, independent of TNF-α and IGF-1 treatment, all cells transfected with HDAC10 selective siRNA showed significant decrease in EZH2 expression. The apparent role of EZH2 in autophagy and its inhibition in absence of HDAC10 suggests its dependence on HDAC10 in activating autophagy related pathways, in VSMCs. When compared between control and HDAC10 knockdown cells that were treated with or without TNF-α and/or IGF-1, little to no apparent change in the expression of DNMT1 was observed (Fig 5 middle panel, lanes 1, 2, and 3 were compared with lanes 5, 6 and 7, respectively). However, when compared between control and HDAC10 knockdown cells that were treated with both TNF-α and IGF-1, the expression of DNMT1 was seen significantly decreased in HDAC10 knockdown cells (Fig 5 middle panel, lane 4 compared to lane 8). These results suggest that the combined treatment with TNF-α and IGF-1 confers indirect effects on DNMT1 expression and is presumably mediated through HDAC10.

In proliferative disorders such as cellular hyperplasia/restenosis and cell survival mechanisms such as autophagy, the eventual end effects will reflect on cellular proliferation. To confirm the role of TNF-α and IGF-1 treatment on HDAC2 and HDAC10 mediated proliferation in VSMCs, we conducted cell proliferation assay using VSMCs that were transfected with or without specific siRNAs that target HDAC2 or HDAC10. The transfected cells were then treated with TNF-α and IGF-1 to study their effects on cell proliferation. As seen in Fig 6, TNF-α and IGF-1 treatment drastically enhanced cell proliferation in VSMCs (Control group compared with TNF-α and IGF-1 treatment group). However, TNF-α and IGF-1 treatment did not enhance cell proliferation in HDAC2 or HDAC10 knockdown VSMCs. Further, in both HDAC2 and HDAC10 knockdown cells, the extent of cell proliferation was similar to as seen in control cells. Since HDAC2 expression is known to enhance cell proliferation, its silencing can affect the normal rate of cell proliferation, in-vitro. On the other hand, expression of HDAC10 would initiate cellular autophagy response, so its knockdown can be expected to have little or no effect on cell proliferation. Moreover, because the expression of HDAC10 is dependent on HDAC2, silencing of HDAC2 is anticipated to show similar effects on cell proliferation as seen in HDAC10 knockdown cells.

Our results support the recently published reports and provide novel evidence which shows that the expression of HDAC10 is directly dependent on HDAC2. Further, our findings show that HDAC2 regulates the expression of PCNA, possibly through an indirect mechanism that involves HDAC10 and EZH2 expression, which are required for the initiation of cellular autophagy in VSMCs. The observed results on HDAC10 dependent expression of EZH2, and the fact that both HDAC10 and EZH2 are markers of cellular autophagy, suggests that TNF-α/IGF-1/DNMT1/HDAC2/HDAC10 axis forms a novel epigenetic pathway that activates cellular mechanisms of autophagy in VSMCs.

## Conclusions

Since DNMT1 expression was known to be induced by TNF-α and IGF-1 through at least two different epigenetic pathways, and HDAC2 expression can be induced by treatment with TNF-α, it is very likely that HDAC2 functions upstream in the TNF-α and IGF-1 induced epigenetic signaling. Cumulatively, our results suggest HDAC2 dependent regulation of an epigenetic pathway with HDAC10, DNMT1, EZH2 and PCNA as effector proteins. Based on the results observed here and the functional interaction domains on DNMT1, we speculate that the TNF-α and IGF-1 induced DNMT1 interacts with HDAC2 to promote HDAC10 activity. Subsequently, HDAC10 facilitates PCNA expression to initiate cell proliferation in VSMCs. It is likely that the observed cell proliferation is not exclusively caused by HDAC10 and PCNA alone since HDAC10 primarily favors autophagy mediated cell survival. Although expression of EZH2 was found to be dependent on HDAC2 and HDAC10, considering its known functions in regulating multiple molecular pathways, its precise role in regulating protein mediators that enhance VSMC proliferation is yet to be discerned. The molecular mechanisms presented here supports our previous reports and other published reports on the development of intimal hyperplasia and restenotic lesions. The present findings identify a discrete and essential role of HDAC2 and HDAC10 as epigenetic modulators that drive molecular pathways initiated by TNF-α and IGF-1 in VSMCs. Additional studies are also warranted which can decipher both the direct and indirect epigenetic signaling mechanisms of TNF-α and IGF-1 that affect cellular functions such as cell proliferation and autophagy.

## Supporting information

S1 Fig (JPG)

S2 Fig (JPG)

S3 Fig (JPG)

S4 Fig (JPG)

S5 Fig (JPG)

S6 Fig (JPG)

S1 Raw images (PDF)

S1 File (XLSX)
