# Supplementary figures and images for: Short communication: TNF-α and IGF-1 regulates epigenetic mechanisms of HDAC2 and HDAC10
Source: PLoS One. 2022 Feb 10;17(2):e0263190. doi: 10.1371/journal.pone.0263190 (PMC8830685; doi:10.1371/journal.pone.0263190)

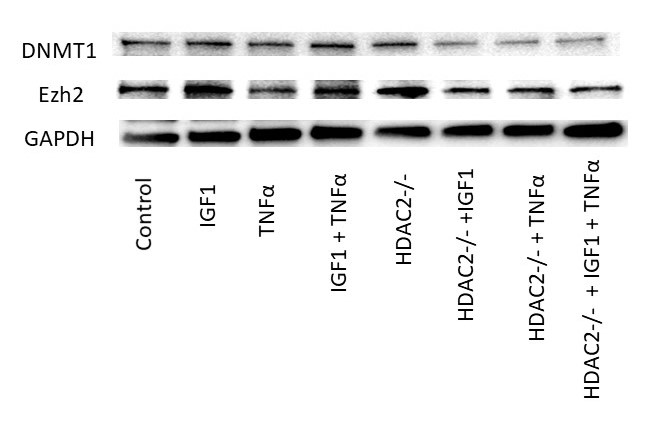

Supplement: S1 Fig — (JPG) [file pone.0263190.s001.JPG]

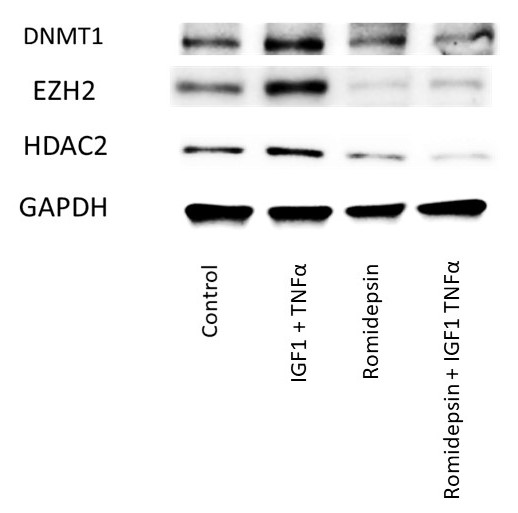

Supplement: S2 Fig — (JPG) [file pone.0263190.s002.JPG]

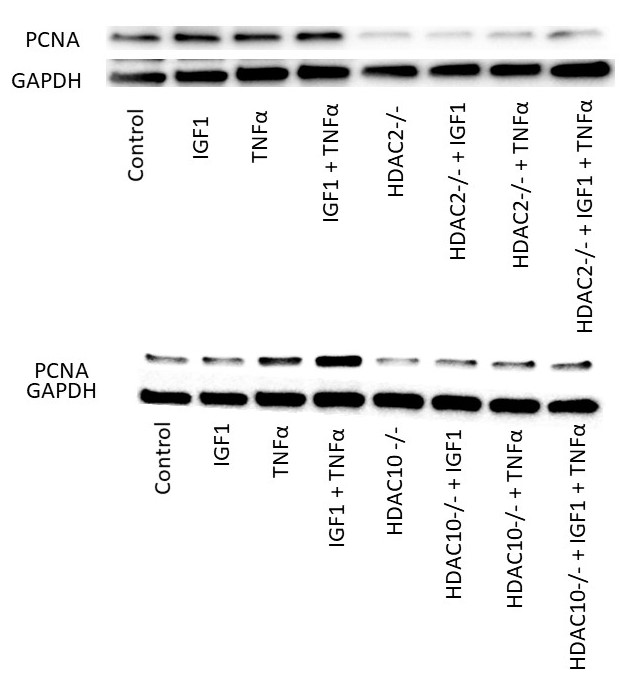

Supplement: S3 Fig — (JPG) [file pone.0263190.s003.JPG]

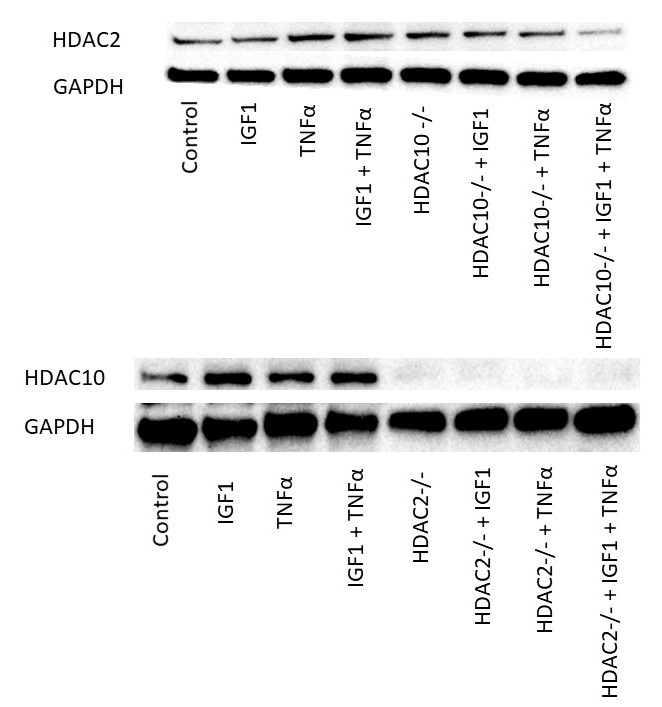

Supplement: S4 Fig — (JPG) [file pone.0263190.s004.JPG]

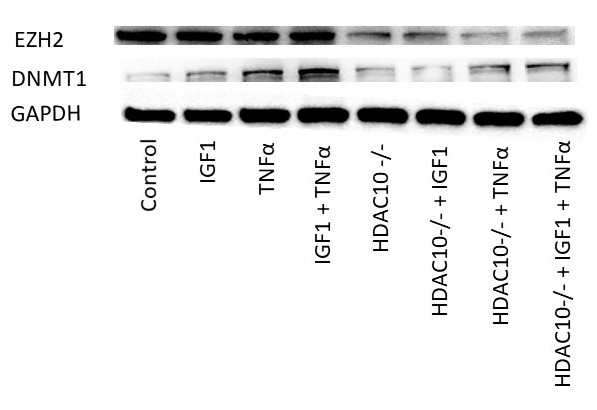

Supplement: S5 Fig — (JPG) [file pone.0263190.s005.JPG]

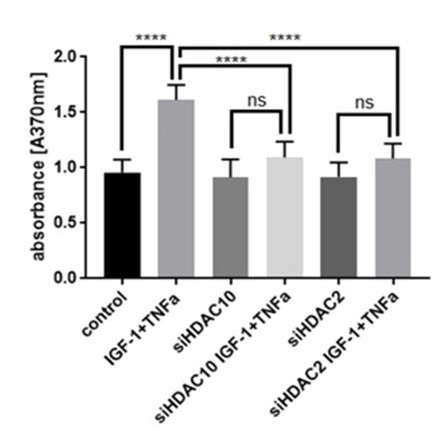

Supplement: S6 Fig — (JPG) [file pone.0263190.s006.JPG]
