# Supplementary material for: Short communication: TNF-α and IGF-1 regulates epigenetic mechanisms of HDAC2 and HDAC10
Source: PLoS One. 2022 Feb 10;17(2):e0263190. doi: 10.1371/journal.pone.0263190 (PMC8830685; doi:10.1371/journal.pone.0263190)
Supplement: S1 Raw images — (PDF) [file pone.0263190.s007.pdf]

Supplemental Fig 1

DNMT1

EZH2

GAPDH

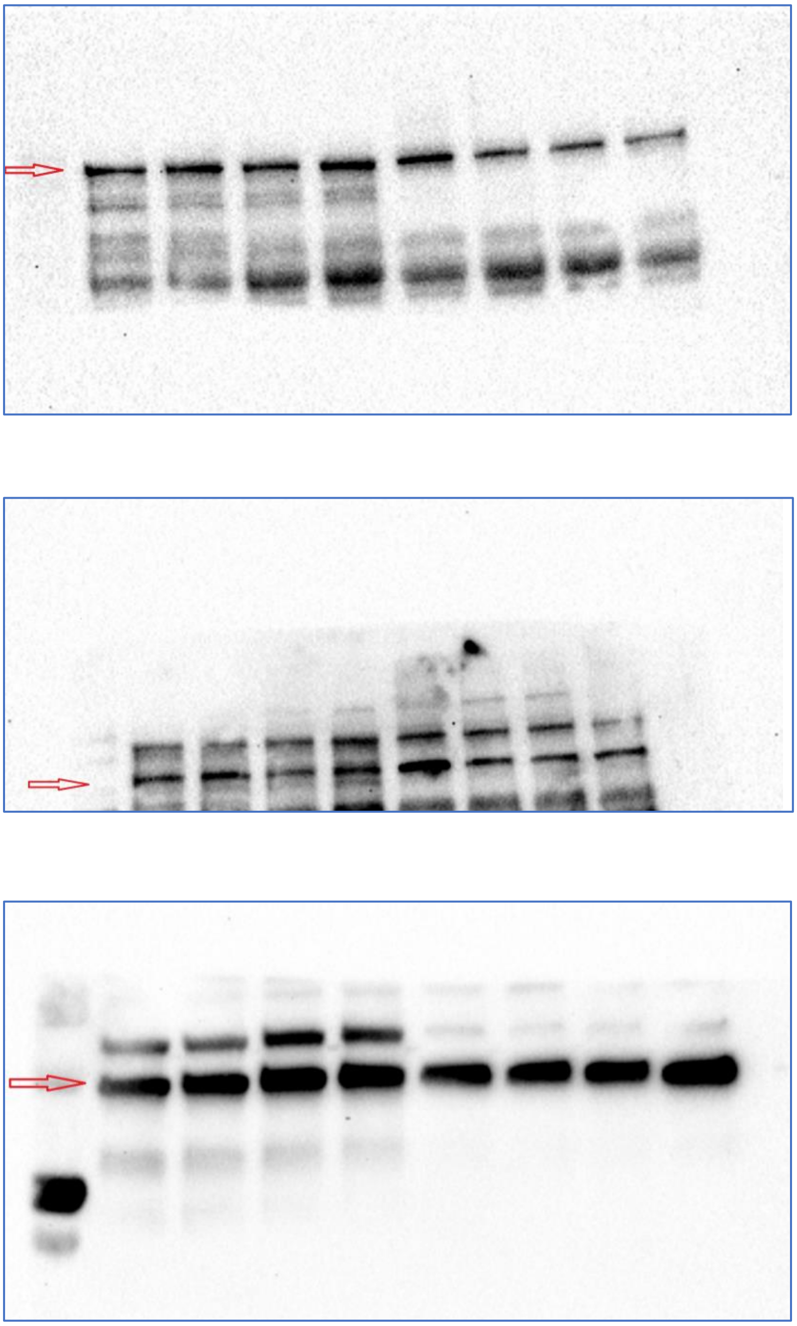

From Left to Right: Mol wt marker (not visible), Control, IGF1 treated, TNFa treated, IGF1 and TNFa treated, HDAC2 siRNA, HDAC2 siRNA IGF1 treated, HDAC2 siRNA TNFa treated, and HDAC1 siRNA with IGF1 and TNFa treated. Arrow indicates expected band size. Note that GAPDH is same for Fig 1, Fig 3a and Fig 4b as they are all HDAC2 siRNA cells but may have different exposure times.

Supplemental Fig 2

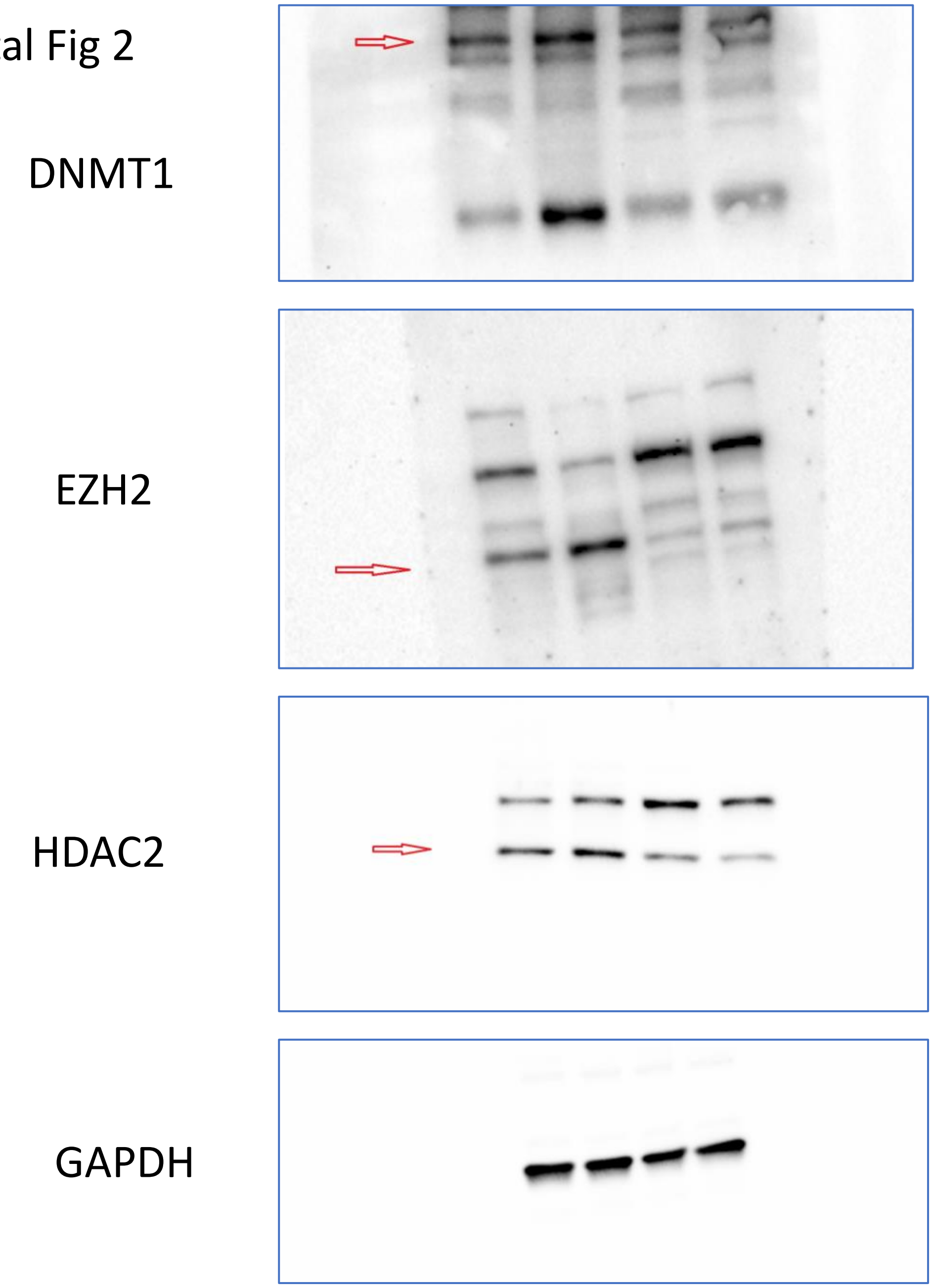

From Left to Right: Mol wt marker (not visible), Control, IGF1 and TNFa treated, Romidepsin treated, Romidepsin with IGF1 and TNFa treated. Arrow indicates expected band size.

Supplemental Fig 3

PCNA

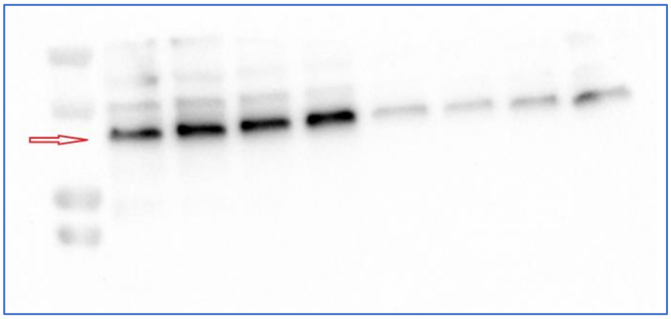

GAPDH
Reused blot

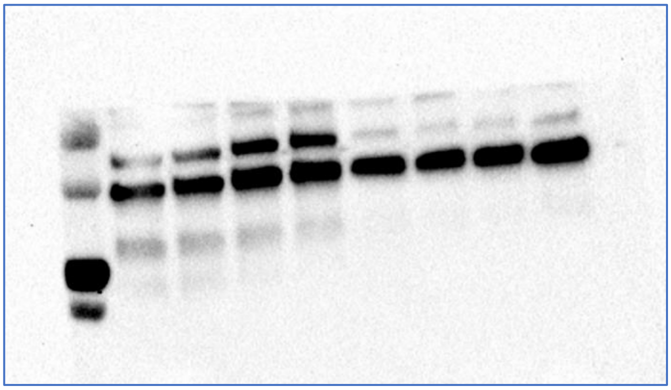

PCNA

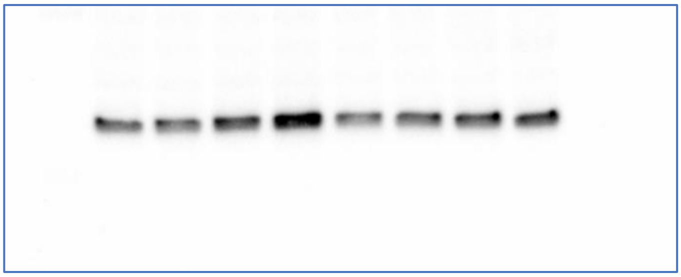

GAPDH

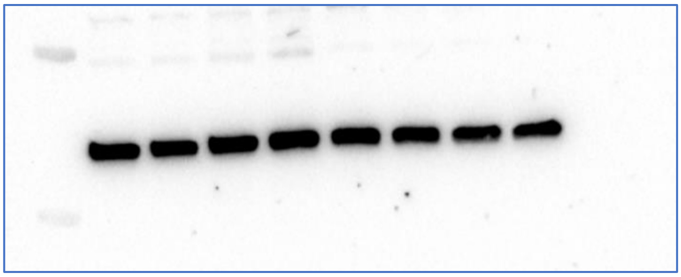

From Left to Right: Mol wt marker (not visible), Control, IGF1 treated, TNFa treated, IGF1 and TNFa treated, HDAC2 (or 10) siRNA, HDAC2 (or 10) siRNA IGF1 treated, HDAC2 (or 10) siRNA TNFa treated, and HDAC2 (or HDAC10) siRNA with IGF1 and TNFa treated. Arrow indicates expected band size. Note that GAPDH is same for Fig 1, Fig 3a and Fig 4b as they are all HDAC2 siRNA cells (reused blot) but may have different exposure times. Also, GAPDH is same for Fig 3b, Fig 4a and Fig 5 as they are all HDAC10 siRNA cells but may have different exposure times.

Supplemental Fig 4

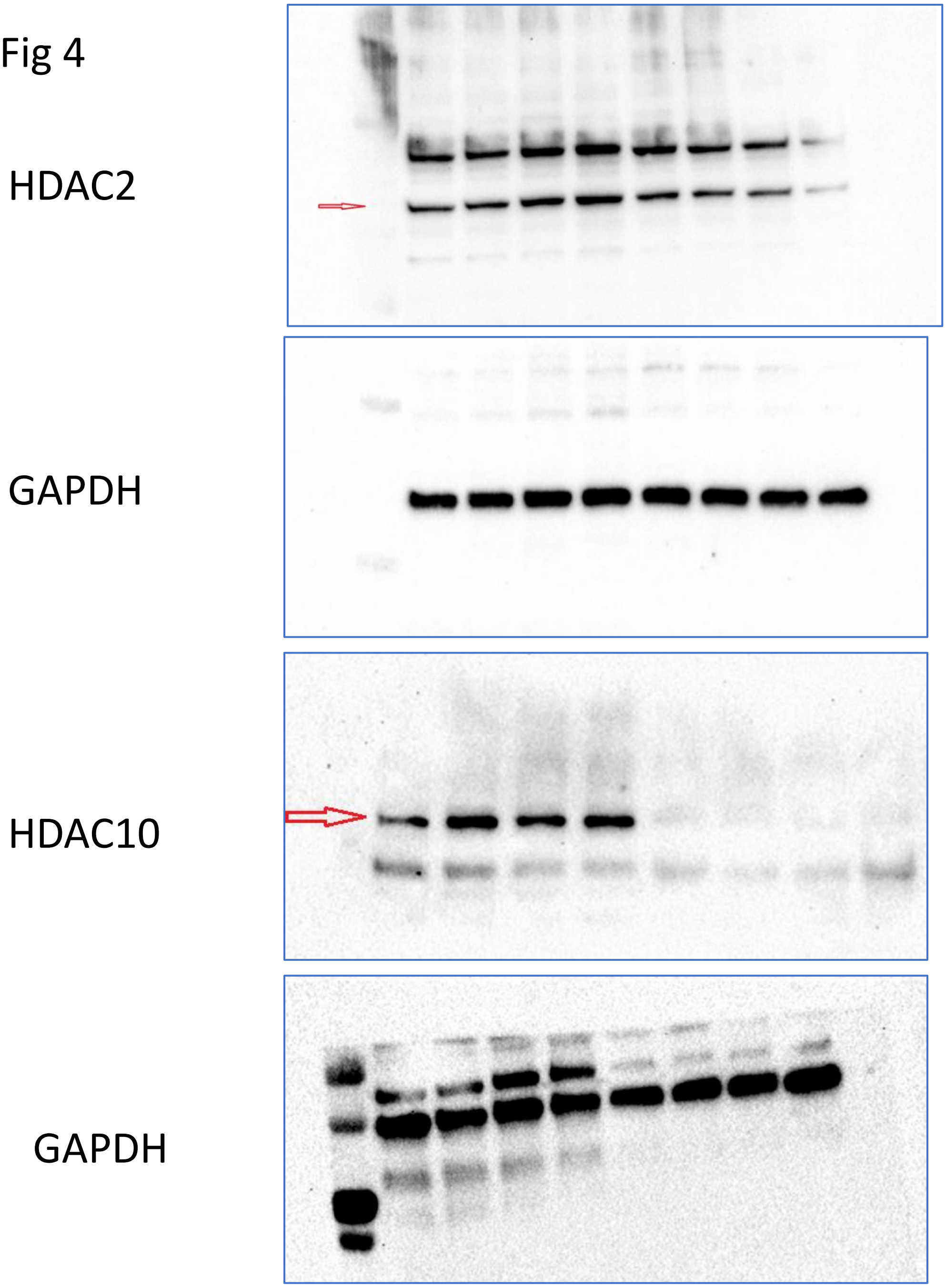

From Left to Right: Mol wt marker (not visible), Control, IGF1 treated, TNFa treated, IGF1 and TNFa treated, HDAC2 (or 10) siRNA, HDAC2 (or 10) siRNA IGF1 treated, HDAC10 (or 2) siRNA TNFa treated, and HDAC10 (or HDAC2) siRNA with IGF1 and TNFa treated. Arrow indicates expected band size. Note that GAPDH is same for Fig 1, Fig 3a and Fig 4b as they are all HDAC2 siRNA cells but may have different exposure times. Also, GAPDH is same for Fig 3b, Fig 4a and Fig 5 as they are all HDAC10 siRNA cells but may have different exposure times.

Supplemental Fig 5

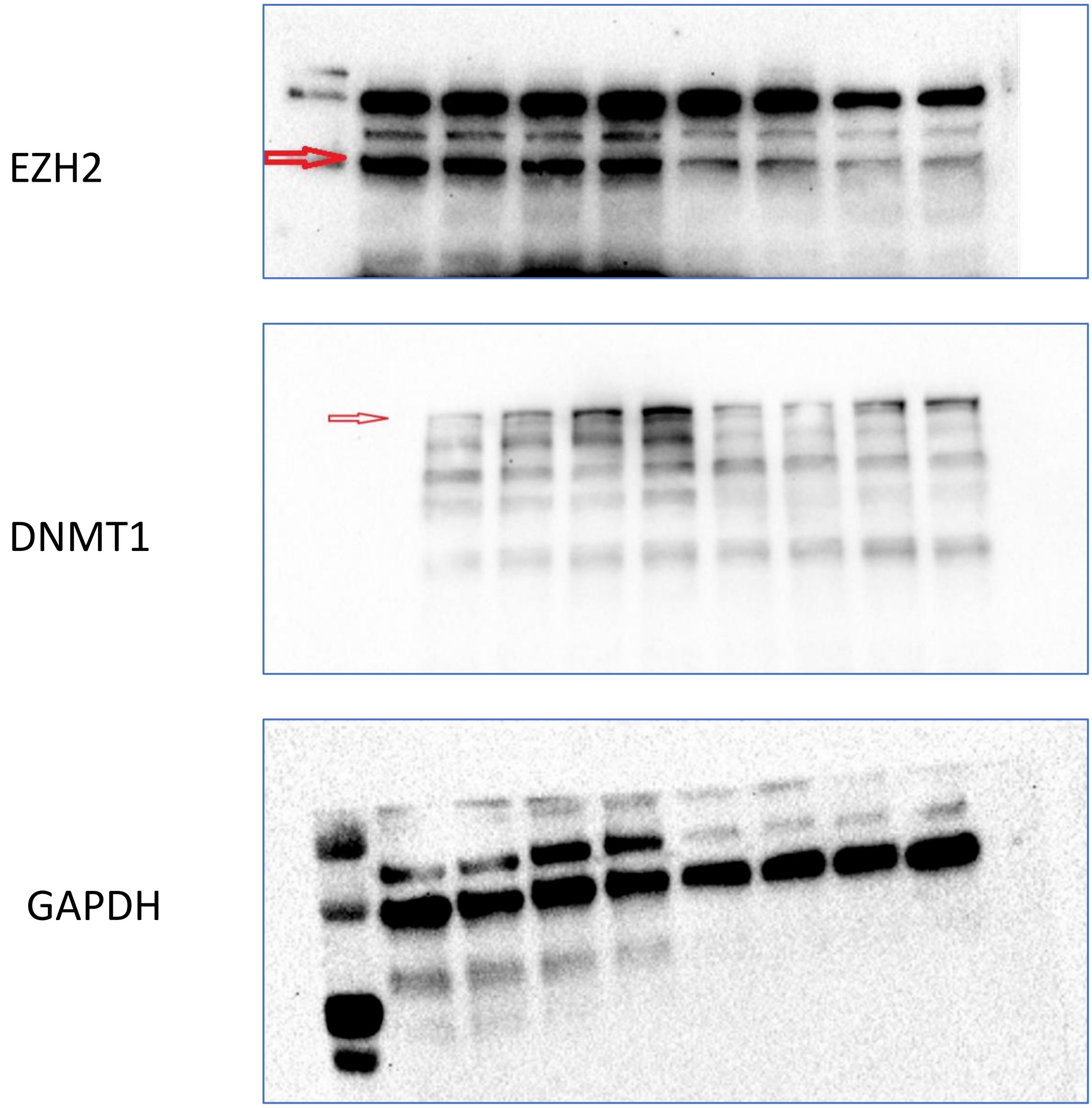

From Left to Right: Mol wt marker (not visible), Control, IGF1 treated, TNFa treated, IGF1 and TNFa treated, HDAC10 siRNA, siRNA IGF1 treated, HDAC10 siRNA TNFa treated, and HDAC10 siRNA with IGF1 and TNFa treated. Arrow indicates expected band size. Note that GAPDH is same for Fig 3b, Fig 4a and Fig 5 as they are all HDAC10 siRNA cells but may have different exposure times.
